# Adaptive capacity in the foundation tree species *Populus fremontii*: implications for resilience to climate change and non-native species invasion in the American Southwest

**DOI:** 10.1093/conphys/coaa061

**Published:** 2020-07-13

**Authors:** Kevin R Hultine, Gerard J Allan, Davis Blasini, Helen M Bothwell, Abraham Cadmus, Hillary F Cooper, Chris E Doughty, Catherine A Gehring, Alicyn R Gitlin, Kevin C Grady, Julia B Hull, Arthur R Keith, Dan F Koepke, Lisa Markovchick, Jackie M Corbin Parker, Temuulen T Sankey, Thomas G Whitham

**Affiliations:** 1 Department of Research, Conservation and Collections, Desert Botanical Garden, 1201 North Galvin Parkway, Phoenix, AZ 85008, USA; 2 Department of Biological Sciences and Merriam-Powell Center for Environmental Research, Northern Arizona University, 617 South Beaver Drive, Flagstaff, AZ 86011, USA; 3 School of Life Sciences, Arizona State University, 427 East Tyler Mall, Tempe, AZ 85281, USA; 4 Research School of Biology, Australian National University, 134 Linnaeus Way, Canberra ACT2601, Australia; 5 School of Informatics, Computing, and Cyber Systems, Northern Arizona University, 1295 South Knoles Drive, Flagstaff, AZ 86011, USA; 6 Sierra Club – Grand Canyon Chapter, 514 West Roosevelt Street, Phoenix, AZ 85003, USA; 7 School of Forestry, Northern Arizona University, East Pine Knoll Drive, Flagstaff, AZ 86011, USA

**Keywords:** Canopy thermal regulation, hybridization, local adaptation, mycorrhizal mutualists, phenotypic plasticity, unmanned airborne remote sensing

## Abstract

*Populus fremontii* (Fremont cottonwood) is recognized as one of the most important foundation tree species in the southwestern USA and northern Mexico because of its ability to structure communities across multiple trophic levels, drive ecosystem processes and influence biodiversity via genetic-based functional trait variation. However, the areal extent of *P. fremontii* cover has declined dramatically over the last century due to the effects of surface water diversions, non-native species invasions and more recently climate change. Consequently, *P. fremontii* gallery forests are considered amongst the most threatened forest types in North America. In this paper, we unify four conceptual areas of genes to ecosystems research related to *P. fremontii’s* capacity to survive or even thrive under current and future environmental conditions: (i) hydraulic function related to canopy thermal regulation during heat waves; (ii) mycorrhizal mutualists in relation to resiliency to climate change and invasion by the non-native tree/shrub, *Tamarix*; (iii) phenotypic plasticity as a mechanism for coping with rapid changes in climate; and (iv) hybridization between *P. fremontii* and other closely related *Populus* species where enhanced vigour of hybrids may preserve the foundational capacity of *Populus* in the face of environmental change. We also discuss opportunities to scale these conceptual areas from genes to the ecosystem level via remote sensing. We anticipate that the exploration of these conceptual areas of research will facilitate solutions to climate change with a foundation species that is recognized as being critically important for biodiversity conservation and could serve as a model for adaptive management of arid regions in the southwestern USA and around the world.

## Introduction

A foundation species is often defined as one that creates environmental conditions that are necessary for the survival of other species by stabilizing fundamental ecosystem processes (Ellison *et al.*, 2005). Almost all foundation species are locally abundant, regionally common and are most often trees with morphological and physiological characteristics that define forest structure, microclimate and ecohydrology. However, foundation tree species are declining throughout the world due to a combination of interactive global change factors including changes in climate, outbreaks of pests and pathogens, logging and alterations in ecosystem hydrology (Ellison *et al*., 2005; Vörösmarty *et al*.**,** 2010). In many ecosystems, community structure and ecosystem processes are largely controlled by a single foundation species (Ellison *et al*., 2005; Peters and Yao, 2012). Therefore, the loss of any one foundation species could have dramatic cascading impacts on a broad range of ecosystem services from forested landscapes, including nutrient and water cycles, food webs, biodiversity and habitat for threatened and endangered species.

*Populus fremontii*, S. Wats. (Fremont cottonwood) is a dominant riparian tree that occupies a broad climatic range across the southwestern USA. It is also recognized as a critically important foundation species in the southwest (Whitham *et al*., 2008) because of its ability to structure communities across multiple trophic levels, drive ecosystem processes (Whitham *et al*., 2008) and influence biodiversity via genetic-based functional trait variation (e.g. Schweitzer *et al*., 2008; Lamit *et al*., 2014, 2015, 2016; Keith *et al*., 2017). However, drought and altered flow regimes combined with land use changes (Merritt and Poff, 2010) have resulted in a 97% decline of pre-20^th^ century habitat (Noss *et al*., 1995). Consequently, *P. fremontii* gallery forests, and more broadly, riparian ecosystems—which support a disproportionately high wildlife biodiversity in arid regions (Poff *et al*., 2012)—are particularly susceptible to both climate change (Gitlin *et al*., 2006) and invasive species (Merritt and Poff, 2010). The little remaining riparian habitat is faced with ongoing climate stress as the southwestern USA is becoming warmer and drier, experiencing decreased river flows, decreased soil moisture and increased evaporative demand (Garfin *et al*., 2013). Decreases in plant available water coupled with increases in the duration and frequency of episodic heat waves have resulted in recent *P. fremontii* die-offs along many major river reaches throughout the Southwest (Gitlin *et al*., 2006).

Compounding these climate-induced reductions in riparian habitat is the successful spread and establishment of *Tamarix* (a.k.a. tamarisk or salt cedar) into riparian areas throughout the western USA. Habitat once dominated by native *P. fremontii* and other *Populus* species has been replaced by *T. ramosissima* and/or *T. chinensis* and their hybrids, with the highest densities along dammed river systems (Busch and Smith, 1995; Merritt and Poff, 2010). *Tamarix* invasion has resulted in extensive monocultures along many major river corridors and tributaries. Once *Tamarix* is established along a river reach, it often increases soil salinity (Taylor *et al*., 1999; Merritt and Shafroth, 2012), alters hydrology (Graf, 1978) and reduces native vegetation cover (Busch and Smith, 1995) and wildlife communities (Ellis *et al*., 1997; Bateman *et al*., 2013). *Tamarix* also disrupts belowground mycorrhizal fungal communities upon which native trees like *P. fremontii* depend (Meinhardt and Gehring, 2012), leaving behind a legacy of low mycorrhizal abundance even when it is removed (Hultine *et al*., 2015). Despite considerable efforts to control *Tamarix* through biocontrol and mechanical removal campaigns (Hultine *et al*., 2010; González *et al*., 2018), there has been a continued decline in native riparian habitat with deleterious impacts on ecosystem function, biodiversity (Harms and Hiebert, 2006) and even agriculture (Zavaleta, 2000).

**Figure 1:**
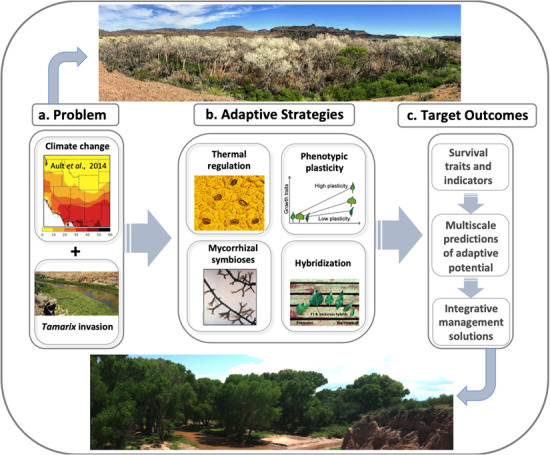
Overall approach to investigating adaptive survival strategies in *Populus fremontii* as responses to climate change and non-native species invasion. (**a**) Climate change and *Tamarix* invasion that can synergistically result in rapid mortality of *P. fremontii* forests (e.g. upper photo of deceased *P. fremontii* trees along the lower Bill Williams River in west central Arizona in 2017—photo credit: Hillary Cooper). (**b**) Four adaptive strategies that are subject to climate change and invasive species as selective agents. (**c**) Target outcomes of integrated management approaches that amplify adaptive potential of *P. fremontii* and riparian ecosystem structure and function (e.g. lower photo of vibrant *P. fremontii* gallery forest along the upper San Pedro River in southeastern Arizona—photo credit: Kevin Hultine).

This review highlights the primary threats to *P. fremontii* and its capacity to serve as an important foundation species across its range in the face of climate change and the presence of non-native *Tamarix*. We start by reviewing the population genomics of *P. fremontii* and identify the key adaptive traits that underpin its current success across its range. Our review synthesizes previous investigations using experimental common gardens and greenhouse experiments to evaluate the expression of genetically based traits that largely govern resource uptake and stress tolerance. Our review will specifically focus on two key mechanisms to cope with the primary challenges of global environmental changes: (i) the expression of leaf/canopy traits required to balance trade-offs between minimizing plant hydraulic dysfunction and minimizing canopy thermal stress, and (ii) the maintenance of mycorrhizal symbionts in the presence of climate change and *Tamarix* presence that can synergistically disrupt mycorrhizal associations (Fig. 1). We argue that traits that best optimize the balance between water loss and canopy thermal regulation during climate stress, and traits that maximize mycorrhizal associations in *Tamarix* soil legacies are likely to be under some of the most significant current and future selection pressures. We therefore hypothesize that the expression of these traits will most likely be identified in (i) genotypes with the highest plasticity in trait expression across environmental conditions such that survival and adaptation to climate stress and *Tamarix* presence correlates with plasticity (Fig. 1) and (ii) hybrids between *P. fremontii* and other closely related *Populus* species yielding genotypes that are more adaptable to climate stress and *Tamarix* presence than either parental species. We anticipate that future restoration efforts of *P. fremontii* gallery forests will require selection of naturally occurring populations and genotypes in the wild and breeding programmes targeting a suite of traits that can best maximize resource use efficiency during periods of resource limitations and maximize resource uptake efficiency during brief resource pulses (Fig. 1). We suggest that high spatial and spectral resolution remote sensing data and associated methods can inform restoration by detecting key traits in the common gardens and upscaling trait detection to the landscape and ecosystem scales.

**Figure 2:**
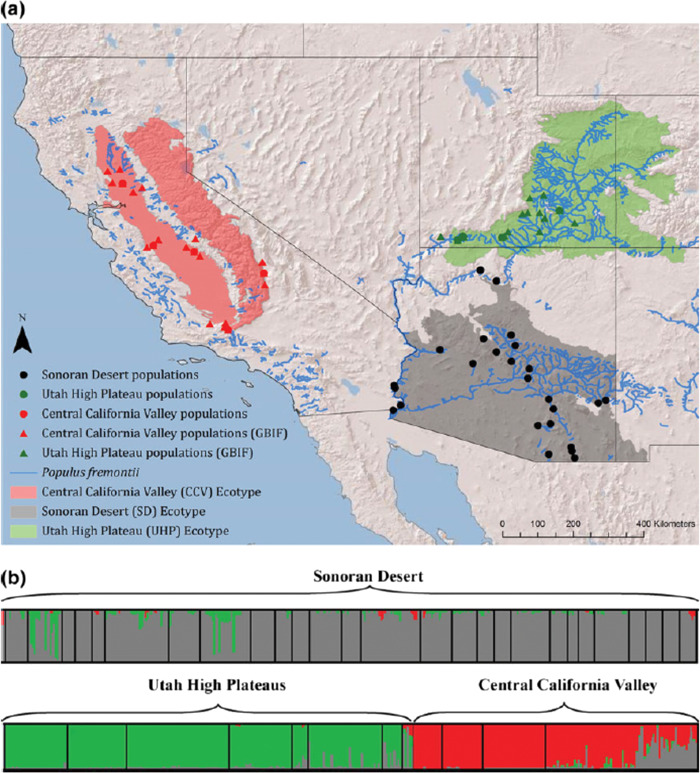
From Ikeda *et al*. (2017). (**a**) Distribution of *P. fremontii* ecotypes. Genetic collections [circles, from Cushman *et al*. (2014)] and Global Biodiversity Information Facility (GBIF) occurrence locations (triangles) were used to construct ecological niche models for the three ecotypes: Central California Valley (red), Utah High Plateau (green) and Sonoran Desert (grey). (**b**) STRUCTURE diagram (Pritchard *et al*., 2000) showing *P. fremontii* population genetic structure. Each bar represents an individual tree; different colours indicate probability of belonging to a given genetic group.

**Figure 3:**
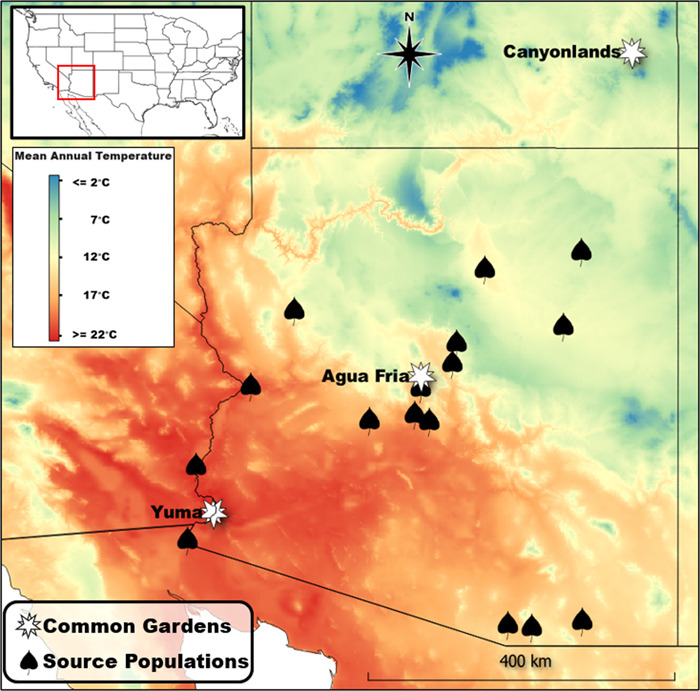
Map showing locations of *P. fremontii* source populations and network of reciprocal common garden mean annual temperatures and locations distributed across a broad climate, elevational and latitudinal gradient.

## *Populus fremontii* population genomics and the role of experimental common gardens

*P. fremontii* exhibits substantial genetic variation throughout its range (Cushman *et al*. 2014), which encompasses broad environmental gradients extending from Mexico, Arizona and California in the south and reaching northward into Nevada and northern Utah (Eckenwalder, 1977). Based on extensive field collections, Cushman *et al*. (2014) found that *P. fremontii* is strongly differentiated into three genetic groups (Fig. 2b), and genetic variation amongst populations is primarily driven by climate gradients and connectivity along river networks. Ikeda *et al.* (2017) utilized this genetic data coupled with additional occurrence records from the Global Biodiversity Information Facility (GBIF) to generate ecological niche models, which defined three climatically differentiated ecotypes comprising the Utah High Plateau (UHP), Sonoran Desert (SD) and California Central Valley (CCV) (Fig. 2a). Ikeda *et al*. (2017) also demonstrated that the inclusion of genetic marker data improved the predictive power of ecological niche models by 12-fold when compared to models that included no population genetic information, leading to more accurate forecasting of changes in *P. fremontii’s* distribution as a function of climate change. More recently, Bothwell *et al*. (unpublished data) examined genomic variation and structure in *P. fremontii* based on restriction site associated DNA sequencing (RADseq). Using ~9000 single nucleotide polymorphisms (SNPs), they found additional support for the three main ecoregions, as defined by Ikeda *et al.* (2017) showing that genetic structure in *P. fremontii* strongly correlates with variation in three primary environmental variables (minimum temperature of the coldest month, precipitation seasonality and mean temperature of the coldest quarter of the year). Together, these data support a hypothesis of strong niche differentiation as suggested by Ikeda *et al*. (2017) and provide the basis for investigating how environmental variation has shaped genetic variation and structure in *P. fremontii*.

One powerful tool for investigating genetic variation, patterns of local adaptation and phenotypic plasticity is through the use of experimental common gardens. One example of a successfully constructed reciprocal common garden network is with *P. fremontii* in the southwestern USA (Fig. 3). The gardens were originally established in 2014 from cuttings collected from 12 genotypes per 16 source populations representing two of the three ecoregions defined by Ikeda *et al*. (2017; Fig. 2), including the UHP and SD ecoregions (Fig. 3). Each garden consists of over 4000 trees planted in four replicated blocks. The three gardens span an elevation gradient of almost 2000 m encompassing an extensive range of temperature extremes experienced by *P. fremontii* (Cooper *et al*., 2019). Mean annual temperature ranges from 10.7°C at the highest elevation garden near Canyonlands, UT, to 17.2°C at the mid-elevation garden north of Phoenix, AZ, to 22.8°C at the low-elevation garden near Yuma, AZ (Fig. 3). The summation of the three reciprocal gardens provides a robust tool for studying the impacts of climate change on phenotypic expression, productivity and fitness of *P. fremontii* in ways that were previously impossible at the landscape scale.

**Figure 4:**
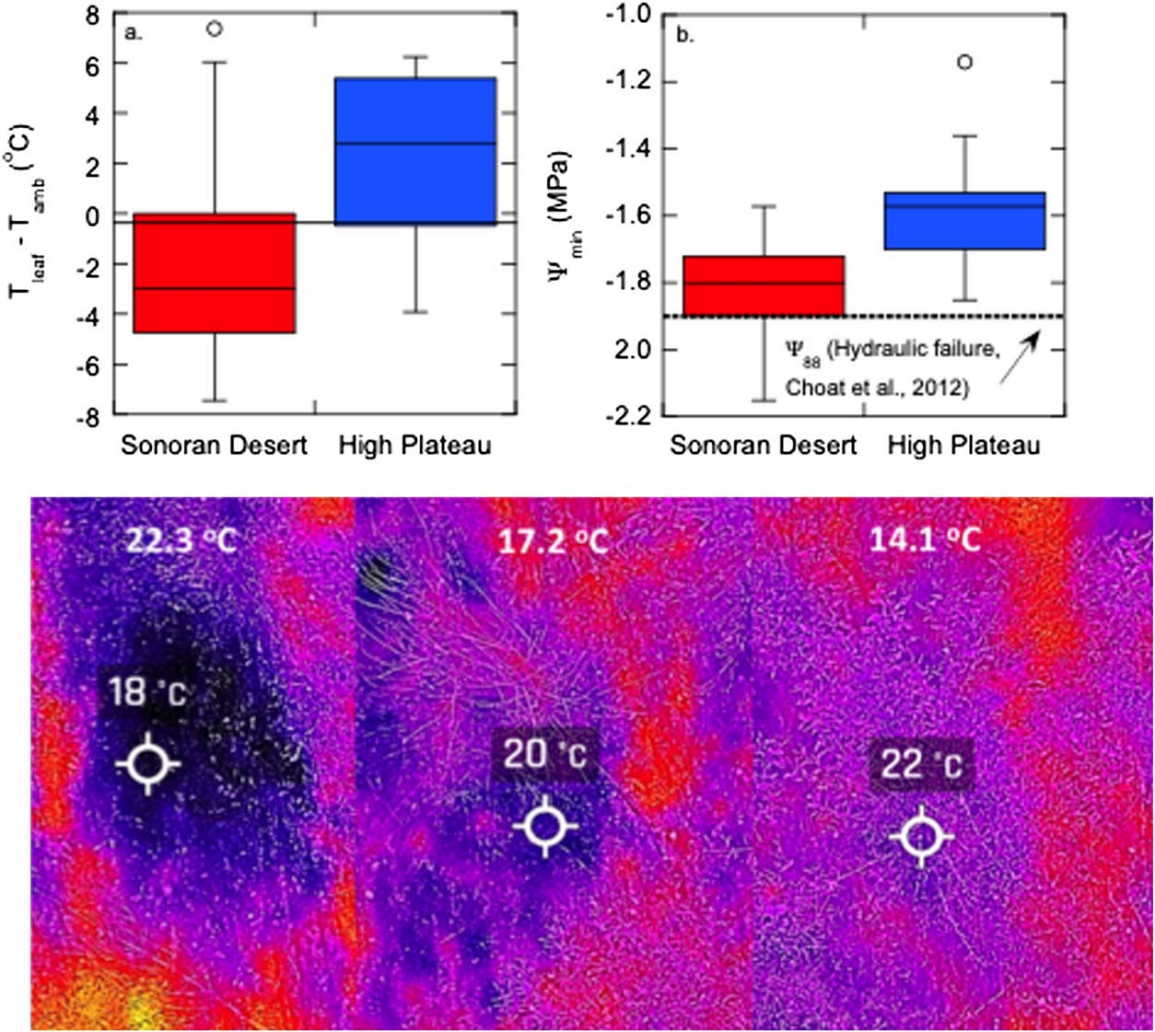
Contrasts in leaf temperature and leaf water potentials between high- and low-elevation *Populus fremontii* genotypes measured in the mid-elevation common garden (Figure 3). (**a**) Box and whisker plots showing the median, 25th and 75th percentiles (box plots) and 10th and 90th percentiles (error bars) of leaf surface temperatures (*T*_leaf_) subtracted from ambient temperature (*T*_amb_). (**b**) Box and whisker plots of minimum daily leaf water potentials (Ψ_min_) measured monthly over the growing season with a Scholander type pressure chamber. Lower panels represent canopy temperatures measured with a thermal camera on three genotypes sourced from three populations varying in mean annual temperatures (shown in white text).

## Trade-offs between thermal regulation and hydraulic risk

Episodic heat waves that are increasing in duration, frequency and intensity will likely amplify thermal stress, mortality and shifts in hydraulic trait expression. For many plant genotypes, especially those occurring on the warm edge of a species distribution, canopy thermal regulation is critical for maintaining leaf carbon balance. Extreme thermal stress not only increases both mitochondrial and photorespiration (at least in C3 plants) but can also irreversibly damage the electron transport capacity of Photosystem II. Thus, exposure to heat stress acts as a strong agent of selection in a broad range of taxa in terms of phenology, hydraulic architecture, xylem anatomy and stomatal regulation. In environments where thermal loads approach an upper limit above which leaf function is impaired (*T*_crit_), plants must transpire water to evaporatively cool leaves below *T*_crit_ (i.e. *T*_leaf_ < *T*_crit_). Recent evidence indicates that leaf conductance in some species increases when leaf temperatures rise above 40°C, thereby increasing the difference between *T*_leaf_ < *T*_crit_ (Slot *et al*., 2016). In some cases, leaf conductance may increase independent of changes in net photosynthesis, indicating there are alternative water-use strategies in some plants other than maximizing carbon gain for a fixed level of stomatal conductance (Urban *et al*., 2017; Drake *et al*., 2018; Aparecido *et al*., 2020). However, there is an inherent hydraulic risk of maintaining *T*_leaf_ < *T*_crit_ under hot and dry conditions in that leaf conductance could drop plant water potential below a critical threshold (Ψ_crit_). Therefore, plants exposed to extreme thermal conditions must optimize traits to balance canopy thermal regulation with plant hydraulic limits.

On the warm edge of its distribution, *P. fremontii* is exposed to some of the warmest mean annual temperatures in North America, often approaching 50°C in midsummer—temperatures that could far exceed *T*_crit_. A recent common garden experiment conducted at the mid-elevation common garden (Fig. 3) revealed that chronic exposure to intense heat waves could impose strong selection pressures on *P. fremontii* to maximize canopy thermal regulation via a suite of hydraulic strategies (Hultine *et al*., 2020). Genotypes sourced from the extremely warm SD ecoregion had midday leaf temperatures in midsummer that were on average 2.0°C (SE ± 0.58) below ambient temperature; while genotypes sourced from the higher elevation, cooler UHP ecoregion had leaf temperatures that were on average 2.1°C (SE ± 0.67) above ambient temperature (Fig. 4a). The cooler leaf temperatures corresponded with the warm-adapted, SD ecotypes having a 35% higher midday leaf transpiration rate (*E*) relative to the UHP ecotypes, resulting in substantially greater leaf evaporative cooling (Hultine *et al*., 2020). Although predawn leaf water potentials (Ψ_pd_) were similar between the two ecotypes, minimum leaf water potentials (Ψ_min_) during midday were on average 0.2 MPa lower in the SD ecotypes (Fig. 4b). The higher leaf *E* coupled with the lower Ψ_md_ suggests that the SD ecotypes have a reduced stomatal control over plant water potential than the UHP ecotypes perhaps as a consequence of having to cope with extreme thermal stress during midsummer. However, a reduced stomatal control over plant water potential is an inherently risky hydraulic strategy, illustrated in Fig. 4b. In many SD genotypes, Ψ_min_ approached**,** or even fell below**,** the xylem pressure (−1.88 MPa) at which near complete hydraulic failure occurs in *P. fremontii*—defined as when the loss of maximum xylem conductivity reaches 88% (Ψ_88_: Choat *et al*., 2012). Conversely, Ψ_min_ in the UHP genotypes never fell below the Ψ_88_ threshold (Fig. 3b), indicating that UHP genotypes have not been selected to take on the same ‘risky’ evaporative cooling strategy as the Sonoran Desert (SD) genotypes.

In order to mitigate the risks of hydraulic failure while maintaining *T*_leaf_ < *T*_crit_ during midsummer heat waves, SD genotypes must optimize a suite of traits related to phenology, hydraulic architecture and xylem function. For example, leaf flush in the low and mid-elevation common gardens occurred as much as 2 months earlier in warm-adapted compared to cold-adapted genotypes (Cooper *et al*., 2019). The earlier leaf flush may be necessary to optimize photosynthetic carbon balance prior to midsummer when the difference between diurnal CO_2_ flux via photosynthesis and CO_2_ efflux via leaf respiration approaches zero due to heat exposure. In order to optimize leaf evaporative cooling, plants adapted to hot environments should also be constructed such that they maximize the supply of water from the soil and vascular system relative to the demand for water via transpiring leaves. (Martinez-Vilalta *et al*., 2009). At the mid-elevation garden, genotypes from the warmer SD ecoregion maintained a 38% higher sapwood area to whole-canopy leaf area ratio relative to cooler UHP ecoregion. Similarly, the same SD genotypes at the mid-elevation garden displayed a 89% higher petiole lumen area to cross-section petiole area fraction—a trait that reflects the maximum water conducting capacity of the petiole—to leaf surface area ratio (Blasini *et al*., unpublished data). Moreover, maximum rooting depth of riparian taxa, including *P. fremontii* is correlated with hydrologic regime (Stromberg, 2013). Experimental evidence indicates ephemeral river reaches are comprised of *P. fremontii* genotypes with deeper roots and larger root area to leaf area ratios than *P. fremontii* genotypes along perennially flowing river reaches (Cadmus *et al*., unpublished data). Thus, genotypes adapted to maintaining high daytime leaf-level transpiration fluxes are likely to also maintain higher root area to leaf area ratios.

Whether the expression of hydraulic architecture and leaf cooling traits is enough to overcome progressive warming and subsequent heat waves that are projected over the next century in the southwestern USA and northern Mexico is an open question. Warming temperatures may lead to significant range shifts in low-elevation SD ecotype plants, but potentially without an overall reduction in range distribution relative to other *P. fremontii* ecotypes (Ikeda *et al*., 2017). However, given the potential hydraulic risk of evaporative cooling, even small reductions in available water coupled with extreme thermal stress could lead to rapid episodic mortality events of *P. fremontii* gallery forests (e.g. upper photo of Fig. 1). Restoring and conserving *P. fremontii* gallery forests will therefore require targeted genetic and phenotypic information to overcome projected environmental constraints on riparian ecosystems.

## *P. fremontii* root symbionts: coping with climate change and *Tamarix*

The results of recent reviews convincingly demonstrate that the mycorrhizal fungi associated with the roots of most plants (Brundrett, 2009) can buffer the effects of climate change, including warming temperatures and drought (Kivlin *et al*., 2013; Mohan *et al*., 2014; Augé *et al*., 2015). The importance of mycorrhizal fungi in the context of climate change is not surprising given that they improve host plant access to soil resources in exchange for fixed carbon (Smith and Read, 2008). In fact, there is evidence that mycorrhizal fungi are more beneficial in stressful situations. In the case of drought, the beneficial effects of mycorrhizal fungi are larger when drought is severe than when it is absent or moderate (Pena and Polle, 2013; Augé *et al*., 2015). However, invasive plants can alter soils in ways that negatively affect the mycorrhizal associations of native plants (Bunn *et al*., 2015, Grove *et al*., 2017). Although the mechanisms are not understood, *Tamarix* alters the mycorrhizal fungal abundance and community composition of *P. fremontii,* (Meinhardt and Gehring, 2012), leaving behind a legacy of low mycorrhizal abundance even after it is removed (Hultine *et al*., 2015). Given that most plant species form mycorrhizal associations, experience climate change and compete with exotic species, it is critical to understand if these fungi help plants overcome the physiological limits imposed by climate change, alone, and in conjunction with non-native competitors or their legacy.

Like many plant species in the western United States, *P. fremontii* faces warming temperatures punctuated by extreme drought, along with widespread non-native competitors such as *Tamarix* that negatively affect cottonwood survival as their densities increase (Gitlin *et al.,* 2006). However, *P. fremontii* is unusual in that it simultaneously forms mycorrhizal associations with two major types of mycorrhizal fungi, the ectomycorrhizas (EM) that occur on many woody perennial plants and the arbuscular mycorrhizas (AM) that occur on the majority of grass, crop and herbaceous plants (Brundrett, 2009). This trait has been observed in a small percentage (~7%) of plant families (Teste *et al*., 2019) with many species only having these dual associations when they are young (Brundrett, 2009; Teste *et al*., 2019). However, *P. fremontii*, *P. angustifolia* and their hybrids form dual associations even when sexually mature (Gehring *et al*., 2006). While both AM and EM fungi enhance soil resource uptake, the two types of associations differ in the environments they occupy (Swaty *et al*., 2016, Soudzilovskaia *et al*., 2019), the soil resources they access (Smith and Read, 2008) and their effects on ecosystem properties (Phillips *et al*., 2013; Jo *et al*., 2019, Soudzilovskaia *et al*., 2019). Ectomycorrhizal fungi are more effective than AM fungi in reducing the effects of soil-borne pathogens in woody plants (Laliberté *et al*., 2015), which may contribute to the tendency for AM-associated trees to have negative plant-soil feedbacks, promoting plant diversity, while EM-associated trees tend to have positive plant-soil feedbacks, leading to monotypic stands (Bennett *et al*., 2017). The limited available data indicates that negative feedbacks are uncommon in dually colonized plants (Teste *et al*., 2019). Understanding the dynamics of AM and EM associations in dually colonized plants, particularly foundation species like *P. fremontii*, is thus important not only to the plant species themselves but has cascading effects on the ecosystem.

**Figure 5:**
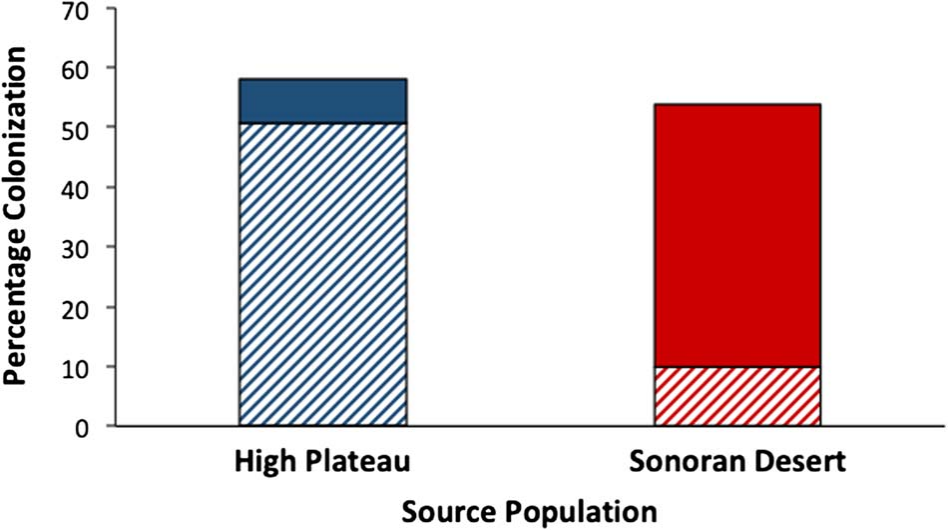
Percent colonization of *P. fremontii* roots by two types of mycorrhizal fungi in mid-elevation common garden, where trees were proximate and conditions similar. AM colonization (solid bars) dominated in trees from the warmer, more arid SD ecoregion, while EM colonization (hatched bars) dominated in trees from the higher, cooler UHP ecoregion. Data represent averages of five root samples per ecoregion. Roots were processed as in Gehring *et al*. (2006).

In the western cottonwoods, *P. angustifolia* and *P. fremontii*, root colonization by AM and EM fungi varies with soil moisture (Gehring *et al*., 2006) and/or cottonwood source population, demonstrating context dependency that could improve cottonwood responsiveness to environmental change. Six weeks of watering in the field during a hot, dry summer altered the dominant mycorrhizal association of juvenile cottonwoods from AM to EM (Gehring *et al*., 2006), a result consistent with studies of oak (Querejeta *et al*., 2009) and with the dominance of AM fungi in warmer, more arid landscapes (Swaty *et al*., 2016). In a mid-elevation common garden, *P. fremontii* from the warmer, more arid SD ecoregion were dominated by AM fungi while those from the UHP ecoregion were dominated by EM fungi (Fig. 5), suggesting a genetic component to dominant mycorrhizal association. Cottonwoods benefit from both AM and EM associations in terms of growth (Meinhardt and Gehring, 2012; Gehring *et al*., 2014), but it is unclear how the combined effects of climate change and *Tamarix* legacy will alter the abundance of these associations or if different cottonwood genotypes will respond similarly. For instance, both AM and EM have been observed to access and move water (e.g. Egerton-Warburton *et al*., 2007) and likely increase moisture retention in the soil (Querejeta *et al*., 2012). However, AM may increase plant water-use efficiency more than EM (e.g. Querejeta *et al*., 2006). Additionally, greenhouse experiments showed that the beneficial effects of mycorrhizas for *P. fremontii* were reduced in the presence of *Tamarix* with greater effects on EM (Meinhardt and Gehring, 2012). Current studies are demonstrating that adding live soil from the rhizosphere of *P. fremontii* from appropriately matched sites without a tamarisk history can improve survival and growth where *Tamarix* has been removed by 30–50% in the greenhouse and in the field (Hull *et al*., unpublished data, Markovchick *et al*., unpublished data). Yet, co-evolution between plants, mycorrhizal fungi and soil (e.g. Johnson *et al*., 1992) can set the stage for inappropriately matched mycorrhizal inoculation to result in neutral to negative plant effects (e.g. Rua *et al*., 2016). Thus, it is not always obvious how to best combine and apply research on mycorrhizal fungi with that on plant hydraulic traits, site adaptation, assisted migration and other management tools.

Further research on combined environmental stressors (drought, heat, invasive species) and interacting management tools is necessary to determine the importance of AM and EM associations to the future resilience of *P. fremontii* as a foundation species. It is also important to understand the future distribution of AM and EM fungi in western US riparian areas because of their distinct influences on ecosystems processes such as carbon storage, nutrient cycling (Phillips *et al*., 2013, Jo *et al*., 2019, Soudzilovskaia *et al*., 2019), water access, plant survival under drought conditions (Egerton-Warburton *et al*., 2007, Teste and Simard, 2008) and plant diversity (Laliberté *et al*., 2015, Teste *et al*., 2019). Inoculation with appropriate mycorrhizal fungi in areas where these fungi are reduced due to the legacy of *Tamarix* invasion could be critical to restoring cottonwoods, their diverse associated aboveground and belowground communities and ecosystem functions.

**Figure 6:**
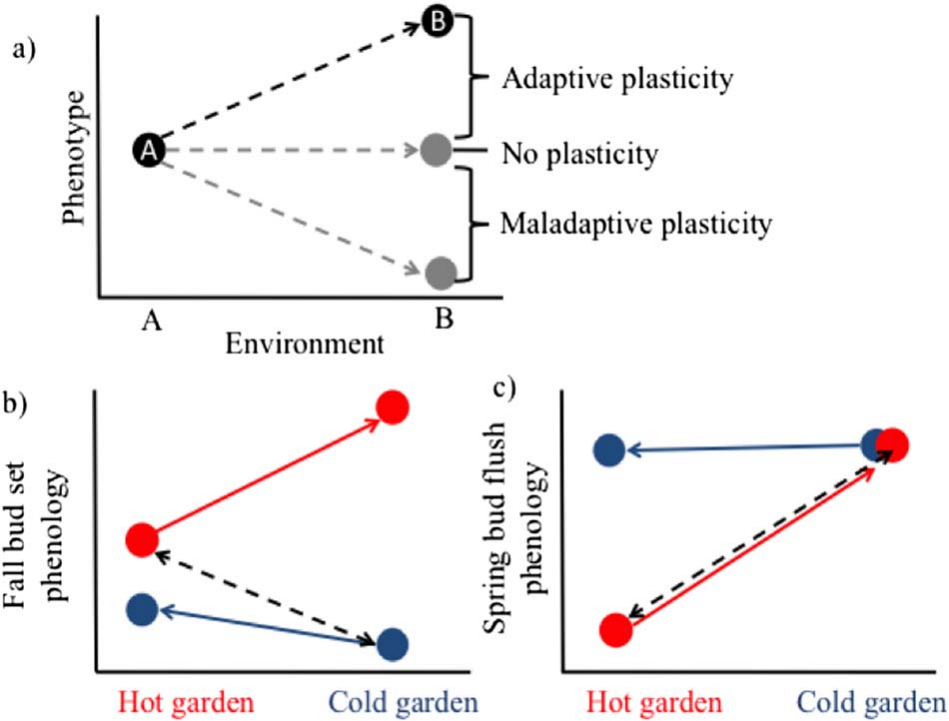
(**a**) Theoretical example of phenotypic plasticity. Environments A and B each have a local genotype (A and B) representing the local optimum phenotype, coloured black. If genotype A is moved to Environment B, it can exhibit adaptive plasticity where the phenotype approaches B. If it matches B’s phenotype (black dashed line), it is perfectly adaptive (Ghalambor *et al*., 2007). If the phenotype does not change, there is no plasticity. If the phenotype changes in the opposite direction as the local optimum, this is considered non-adaptive plasticity. These plastic responses can then impact higher levels of biological organization. (**b**) Fall but set phenology shows non-adaptive plasticity of hot genotypes transferred from the hot garden to the cold garden and slightly adaptive plasticity for cold genotypes transferred from cold to hot gardens. (**c**) Spring bud flush phenology shows highly adaptive plasticity of hot genotypes transferred from hot to cold gardens, and no plasticity for cold genotypes transferred from cold to hot gardens. The dashed black lines show perfectly adaptive plasticity. (b) and (c) adapted from Cooper *et al*. 2019.

## The role of phenotypic plasticity in maximizing resilience to environmental change

Phenotypic plasticity, the capacity of a genotype to express different phenotypes in response to different environments, can facilitate population persistence and influence the strength of selection and degree of genetic adaptation under rapid environmental change (Gibert *et al*., 2019). Plasticity can be expressed in an adaptive direction, where the phenotype shift is towards a local optimum and results in increased plant fitness, or in a non-adaptive direction, with traits shifting away from the local optimum and reducing fitness (Fig. 6a; Ghalambor *et al*., 2007, Gibert *et al*., 2019). Perhaps the most robust examples of phenotypic plasticity in plants revolve around growth and phenology traits and are best examined in multiple common garden experiments where genotypes are replicated across different environments (Franks *et al*., 2014). For example, common garden studies on *P. fremontii* (Fig. 3) show variation in the direction and magnitude of plasticity for two phenology traits, fall bud set and spring bud flush (Cooper *et al.,* 2019). Here, the nature of plasticity that a population expressed (adaptive or non-adaptive) depended on both the trait and the climatic distance between population origin and common garden transplant distance. For example, populations from the hot, southern SD ecoregion exhibited non-adaptive bud set plasticity when they were transferred to the colder, northernmost garden in Utah, while populations from the coldest provenances showed limited adaptive plasticity when transferred into the hottest common garden in southern Arizona (Fig. 6b). Spring bud flush showed varied patterns, with hot populations exhibiting adaptive plasticity and cold populations showing little to no plasticity when grown in the common garden farthest from their source provenance (Fig. 6c). Plasticity in phenology is especially important as adjusting to earlier springs can increase growing season length, which will affect not only long-term growth and competitive ability of *P. fremontii* trees but will also cascade to the broader community of arthropods, microbes, birds, etc. that utilize these plants for habitat and food, as well as ecosystem processes such as carbon and nitrogen cycling (Walker *et al*., 2019). Interestingly, the populations in this study showed a significant correlation between climate of origin and magnitude of plasticity (Cooper *et al.* 2019). Trees from low elevation, hot provenances were much more plastic than trees from cold climates that regularly experience freezing. This suggests inherent limits to plasticity due to the selective abiotic pressures that trees have evolved to withstand.

Common gardens have also been utilized to show significant leaf size and symmetry plasticity in *P. fremontii* as genotypes were transferred to garden climates increasingly different from their home provenance climates (Parker *et al*., in review). Fluctuating leaf asymmetry is commonly used as an indicator of genetic and environmental stress (Wilsey *et al.,* 1998, Beasley *et al*. 2013). Leaf size and symmetry decreased with increasing climate transfer distance to both warmer and colder garden climates, and this non-adaptive plasticity was correlated with lower tree growth and higher mortality (Parker *et al*., in review).

Plasticity can be especially important in preventing long-lived tree species showing high levels of local adaptation from going extinct under rapid climate change events (Franks *et al.,* 2014). The ability of plants to maintain functional water transport systems during periods of extreme climatic stress, such as heat waves or drought, is critical for survival, especially as the frequency of climate extremes is predicted to increase. Phenotypic plasticity has been documented for a range of hydraulic traits in plants subjected to water and temperature stress. For example, increased resistance to cavitation via plastic xylem traits was documented in hybrid popular saplings under drought treatment (Plavcová and Hacke, 2012). Likewise, in years with low water availability, vessel size and density shifted to balance hydraulic conductivity and embolism risk in oaks (Abrantes *et al*., 2013). A recent global review of plasticity in turgor loss point revealed substantial plasticity in wilting point across both wild and crop species (Bartlett *et al.*, 2014). Although there are many studies documenting plasticity in important hydraulic traits in response to environmental change, there may be limits or costs to plasticity due to correlated traits or trade-offs inherent in balancing a functional hydraulic system. For instance, Hultine *et al*. 2020 documented a trade-off in arid-adapted phreatophytic plants, where they can withstand decreases in available groundwater or increases in atmospheric demand (temperature), but not both.

## Hybridization and resiliency to environmental stress

Hybridization has long been recognized as a major evolutionary pathway in the formation of new species (Grant, 1971; Arnold, 1997), and it is estimated that up to 70% of all plants owe their evolutionary origins to ancient and modern hybridization events (Stace, 1987). Some plant groups so readily hybridize (e.g. pines, oaks, eucalypts, cottonwoods) that the total number of species that hybridize within a group is referred to as a syngameon (Grant, 1971). A long history of hybridization events in such species complexes often results in incomplete lineage sorting (Wu *et al.,* 2018) and reticulate evolutionary trees (McKinnon, 2005). This commonality amongst many native syngameons provides evidence for the importance of hybridization as a key evolutionary process that contributes to their continued persistence. Although the conservation of hybrids suffered for many years due to their being considered maladaptive and resulting in the disintegration of pure species (e.g. O’Brien and Mayr, 1991), this issue is much less of a concern with naturally occurring hybrids between native species (Whitham *et al*., 1991, Whitham and Maschinski, 1996). These naturally occurring hybrids often exhibit hybrid vigour and, in some cases, naturally occurring hybrids have been included in the recovery plans of listed species to preserve their evolutionary potential (e.g. U.S. Fish and Wildlife Service, 1995). Eckenwalder (1984) showed that, wherever two *Populus* species come together in the USA and Canada, hybridization occurs, and the geographic distribution of hybrids can be as great as their parental species; e.g. the hybrids between *Populus angustifolia* and *P. fremontii* are found from Mexico to southern Idaho.

Hybrids possess three major attributes that make them especially important in evaluating their potential in conservation and mitigating the impacts of climate change. First, Schweitzer *et al*. (2002) found that both F_1_ (i.e. the first generation cross between two species) and backcross generations (F_1_s can backcross with their parental species to produce a continuum of intermediates between species) can be equally or more fit than the parent taxa. F_1_ hybrids between *P. angustifolia* and *P. fremontii* produced as many viable seeds as *P. angustifolia* (but fewer than *P. fremontii*), and backcross hybrids were equal to both parents. Asexual reproduction through stump sprouting (coppicing) or suckering from root sprouts to form clones was nearly twice as high in hybrids compared to *P. angustifolia* and far greater than *P. fremontii*, which can coppice from stumps, but we have not observed any root sprouting. Similarly, Gom and Rood (1999) found that 5 years after a fire event, 97% of the sprouting trunks and 98% of the root sprouts were from species in the section *Tacamahaca*, which includes *P. angustifolia*, while species in the section *Aigeiros*, which includes *P. fremontii*, accounted for only 3% of the sprouting trunks and 2% of the root sprouts. Associated with these findings they considered the quality of clonal regeneration in *P. fremontii* to be poor with sparse trunk sprouting. They also note that the lack of resprouting by *P. fremontii* allows other invasive clonal species like *Tamarix* to invade following fire. Another study by Schweitzer *et al.* (2002) did not find any resprouting or formation of clones by *P. fremontii* in the field under no fire conditions, but at the same study, sites they found prolific resprouting by *P. angustifolia* and naturally occurring hybrids with *P. fremontii*.

**Figure 7:**
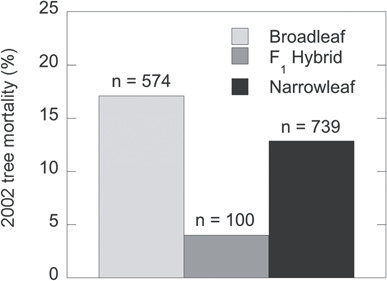
A survey of cottonwood mortality on the Colorado Plateau after the drought year of 2002 revealed higher survival of hybrids than parental species. Forty-six stands were chosen, including 20 narrow-leaf cottonwood stands, 15 Fremont cottonwood stands (broadleaf) and 11 stands in the hybrid zone where upper and lower elevation species are both found along with their F_1_ type hybrids (narrow-leaf *n* = 628 trees; F_1_ type *n* = 100; Fremont (broadleaf) cottonwood *n* = 574). The first 30 standing trees encountered when walking transects perpendicular to the river’s edge were counted and identified based on leaf morphology. Individuals were defined as being >2 m tall and included all resprouting that connected to the main trunk above the ground level. Death was defined as the complete mortality of a single individual, and a tree was considered live if there was evidence of basal resprouting from the trunk. The taxonomic status of dead trees was based upon dried leaves, tree structure and placement in relation to other trees. When a tree could not be clearly identified, it was not included in the survey. In order to capture the effect of the current drought on tree stands, only recently dead trees were counted (i.e. standing trees with intact bark and small diameter branches present). F_1_ type mortality never exceeded 8% for a single stand, while mortality for pure species ranged between 0 and >50% for individual stands. Average F_1_ mortality was 4% and differed significantly from mortality of parental species (*χ*^2^ = 14.889; *P* = 0.0006).

The propensity of hybrids to reproduce asexually may be especially important in a changing environment where drought and altered stream flows due to the damming of rivers can prevent natural recruitment by cottonwoods that require flooding events to create bare mineral sand banks for seedling germination and establishment (Rood *et al*., 2005). Once a hybrid becomes established, it may asexually produce hundreds of progeny that initially receive parental care through their connected root systems, resulting in high survival rates. The clone may persist for 100 to 1000 s of years because regeneration is not dependent on flooding events. The fact that *P. fremontii* does not reproduce asexually via root sprouts could be a major detriment to its long-term survival in the arid Southwest. Even if restoration plantings are successful in altered river systems, lack of natural recruitment will eventually lead to failure of that population (Rood *et al*., 2005; Dixon *et al*., 2012; González *et al*., 2018)**.**

A second major adaptive feature of naturally occurring hybrids is their potential to tolerate drought events better than their parental species. After the Southwest’s record drought in 2002, mortality of F_1_ hybrids in 2003 on the Colorado Plateau was 1/4^th^ and 1/3^rd^ the mortality of *P. fremontii* and *P. angustifolia*, respectively (Fig. 7). Although the specific mechanism(s) that might account for these differences in reproduction and drought tolerance is unknown, molecular genetic analyses show that the hybrids, as expected, can exhibit far greater genetic diversity than their parental species as a result of recombination between the two parental genomes (Whitham *et al*., 1999; Martinsen *et al*., 2001). Such genetic variation may be especially important as it provides a wider range of novel variants for selection to act upon in a changing environment.

Third, naturally occurring hybrids affect communities in diverse ways. (i) They often host the different biota (e.g. arthropod communities) supported by both parental species (review by Whitham *et al*. 1999), which can greatly enhance their conservation importance in riparian habitat that is recognized as a hotspot of biodiversity (Bothwell *et al.,* 2017). Importantly, hybrids also provide critical habitat for some arthropod species found nowhere else that have specifically evolved to live on hybrids (Evans *et al*., 2008, 2012). (ii) Recent studies have also shown that arthropod communities on hybrid versus parental cottonwoods differ significantly in their community phylogenetic structure, suggesting that hybrids are evolutionarily significant units of biodiversity that merit conservation management (Jarvis *et al*., 2017). (iii) Birds respond to the architectural differences of hybrids and some preferentially select hybrids as nesting habitat (Martinsen and Whitham, 1994). (iv) Cottonwoods are preferred forage for beaver (*Castor canadensis*), and their selective foraging can affect the species composition of riparian forests to favour less preferred species and hybrids that resprout prolifically following herbivory*.* In cafeteria choice experiments, Bailey *et al.* (2004) showed that beaver preferred *P. fremontii* more than hybrids and much more than *P. angustifolia*. Furthermore, over a 26-month study in the wild, they showed significantly more *P. fremontii* than hybrids were felled by beaver resulting in a significant shift in stand composition. This shift was driven by the combined effects of selective herbivory as well as the poorer resprouting abilities of *P. fremontii* relative to hybrids and *P. angustifolia* (Gom and Rood, 1999; Schweitzer *et al.,* 2002). This is important because when hybrids are felled by beaver; they generally resprout from both the stump and roots to form clones that can be composed of 100 s of trees. Although McGinley and Whitham (1985) found that while small *P. fremontii* (about 3 m tall) stump sprouted following beaver herbivory, no root sprouting was observed and trees were transformed into shrubs with no formation of clones. At other sites with larger trees, no stump sprouting was observed, and the trees often died following beaver herbivory (Whitham personnel observation). (v) The fungal communities associated with hybrids can alter the performance of the trees themselves as well. For example, *P. angustifolia* cuttings grew significantly larger when inoculated with AM fungal spores from F_1_ hybrids than with spores from *P. fremontii*, or *P. angustifolia* (Gehring *et al.,* 2014). Fungal pathogen communities also differ amongst *P. fremontii*, *P. angustifolia* and their F1 hybrid (Busby *et al.,* 2013).

## Future directions: scaling trait expression from genotypes to ecoregions

The common garden and greenhouse studies described above have identified key genomic, phenotypic and physiological variation that contributes to the capacity of *P. fremontii* and its hybrids to survive in the arid Southwest. Characterizing such variations at the landscape scale and understanding their interactions with climate change overtime requires scaling up measurements and observed relationships from genotypes to ecoregions. Remote sensing provides unique opportunities to measure phenotypic and physiological variation at various temporal and spatial scales. At the individual leaf level, ground-based, handheld spectroradiometer hyperspectral data can provide detailed estimates of plant chemical traits, carbon exchange, reflectance and moisture content. In comparison, unmanned and manned airborne hyperspectral sensors provide similar estimates at the scales of whole tree canopies, field plots or a landscape, whereas spaceborne satellite sensors can provide coarse resolution estimates of total ecosystem net primary productivity, vigor and moisture content at regional, continental and global scales. Taken together, these remote sensing data can provide detailed measurements at individual leaves and canopies, which can then be scaled up to satellite image-derived estimates at broader landscape scales.

Various sensors can be used to detect specific *P. fremontii* traits and their plasticity across environmental gradients. While hyperspectral sensors cover a large range of the electromagnetic spectrum (400–2500 nm) and can, therefore, detect variability in a wide range of plant traits, a suite of other sensors can be used to establish relationship with the hyperspectral measurements in high spatial resolution. In a hierarchical approach, Sankey *et al*. (2017a,b) used ground-based hyperspectral data as well as UAV hyperspectral and lidar data to characterize individual plant species and canopies over small areas covering 1–3-ha areas. Such hyperspectral measurements were further scaled up to include larger areas of 10–30 ha using unmanned aerial vehicle (UAV) multispectral sensors (Sankey *et al*., 2017a,b; Solazzo *et al*., 2018; Elkind *et al*., 2019) that only included four spectral bands centred at the visible and near-infrared regions of the electromagnetic spectrum.

Manned and unmanned airborne multispectral sensors with a few spectral bands can be used at the peak of the growing season to measure total green leaf abundance and biomass at the whole-canopy level across the landscape to determine if these traits differ amongst *P. fremontii* populations and genotypes, between hybrids versus parental species, and between *P. fremontii* trees growing with versus without *Tamarix*. For example, spectral band ratios including normalized difference vegetation index (NDVI) and optimized soil-adjusted vegetation index (OSAVI) from UAV multispectral images have been used to detect differences amongst genotypes within a single tree species (Santini *et al*., 2018). In similar applications for *Populus* trees, individual canopies with significantly greater leaf abundance, biomass, survival rates, NDVI and OSAVI values can be identified to determine genotypes that are best suited to cope with climate change, stress and impacts from *Tamarix* soil legacies. Furthermore, Bedford *et al*. (2018) demonstrated that manned airborne multispectral data can be used across entire river corridors to detect *Tamarix* invasion and subsequent defoliation by an introduced biocontrol agent, the *Tamarix* leaf beetle (Hultine *et al*., 2010). Using manned airborne lidar data in conjunction with multispectral data, Sankey *et al*. (2016) estimated changes in *Tamarix* biomass due to varying rates of defoliation. Similarly, unmanned and manned airborne lidar data (Sankey *et al*., 2013; Shin *et al*., 2018) can be used to detect differences in *Populus* aboveground plant biomass, canopy architecture, height and diameter amongst genotypes and hybrids versus parental species.

Thermal sensors spanning the 8000–12 000-nm spectral range can be used to evaluate patterns of canopy thermal regulation. Specifically, thermal sensors used at the peak of the growing season can measure whole-canopy-scale temperature of individual cottonwood trees (Sankey *et al*., unpublished data). Previous ground-based leaf temperature measurements in common gardens indicate up to 6°C midday difference amongst *P. fremontii* genotypes (Fig. 4), while some currently available thermal sensors are sensitive to differences within 1°C and can detect statistically significant differences amongst genotypes (Santini *et al*., 2019). Our UAV thermal data at a common garden demonstrates that *P. fremontii* populations have significantly different whole canopy-scale mean temperatures (Sankey *et al*., in review). The UAV thermal data further demonstrates that some of the *P. fremontii* genotypes also have significantly different canopy-scale mean temperatures (Sankey *et al*., in review). The temperature measurements can be further used to derive estimates of canopy transpiration and canopy stomatal conductance. While Landsat 8 OLI satellite thermal sensor and ECOSTRESS satellite sensors only provide large pixels of 100-m spatial resolution, the thermal bands can be similarly used to estimate whole *P. fremontii* stand temperature and derive stand-level transpiration and stomatal conductance. Canopy thermal regulation, transpiration and stomatal conductance can be measured across spatial scales by combining high-resolution manned or unmanned airborne images with small spatial extents with coarser resolution satellite images over larger areas.

Many remote sensing techniques can make consistent and repeated measurements at various phenological stages, providing a unique opportunity to detect differences in trait plasticity amongst genotypes. For example, early spring images can be used to detect differences in greening and leaf flush, whereas peak of the growing season images might be used to measure leaf area and survival/mortality to determine if these traits are significantly different amongst genotypes (Sankey *et al*., unpublished data). In such repeated measurements, the high resolution, fine-scale images can be correlated to coarser resolution images covering a much larger area to scale up the specific measurements from genotypes to whole landscapes and ecosystems. In such scaling efforts, the high-resolution images are critical for training and validating the coarser resolution image-based estimates.

## Conclusions: management of *P. fremontii* forests for adaptation and resilience

The capacity for *P. fremontii* to continue supporting diverse biotic communities in aridland riparian areas may depend largely on identifying and exploiting adaptive traits that match future environmental conditions, and our research suggests that optimal genotypes for any given location will depend on trade-offs in their varying capacities to cope with the effects of drought, heat waves, the presence of *Tamarix* and other stressors. In some regions such as the seasonally hot SD, restoration projects will inevitably be limited to genotypes selected for extreme evaporative leaf cooling and locations where groundwater remains stable during the hottest periods of the year. Likewise, successful restoration and maintenance of *P. fremontii* forests may also require active management of plant microbial interactions through inoculation of AM and EM fungi, particularly in areas previously dominated by *Tamarix*. Because environmental conditions of riparian ecosystems are highly dynamic and are expected to experience greater climate extremes in the near future (Garfin *et al.,* 2013), genotypes that express a high degree of plasticity in traits related to frost avoidance, heat avoidance and tolerance to low soil microbial abundance will inevitably be favoured. Finally, restoration ecologists have likely undervalued the potential of using *Populus* hybrids in restoration given evidence suggesting that hybrids are likely to have higher fitness and survivorship compared to parental species when exposed to episodic stress conditions.

Opportunities to better inform restoration of *P. fremontii* and other foundation tree species may be amplified by taking advantage of cutting-edge tools to evaluate the adaptive capacity of plant stress tolerance. Recent advances in *Populus* whole-genome sequencing to evaluate adaptive trait associations (Tuscan *et al*., 2006; Evans *et al.,* 2014), high throughput phenotyping (Araus and Cairns, 2014), hyperspectral imaging of forest canopies (Asner *et al.*, 2017) and coupled fluvial hydrologic/plant hydraulics modelling (Tai *et al.*, 2018) amongst many other advances provide emerging opportunities to rapidly evaluate and predict survivorship and foundational capacity of *P. fremontii* in rapidly changing environmental conditions. Approaches that merge a broad suite of phenotypic traits with process-based models will provide a new way forward to studying and protecting groundwater dependent vegetation, riparian communities and ecosystem processes into the future.

## Funding

This research was supported by the National Science Foundation MacroSystems Biology programme [DEB-1340852 and DEB-1340856], BEE grant DEB-1914433 and MRI-DBI-1126840.
